# Impact of negative signs on therapeutic compliance in patients with schizophrenia

**DOI:** 10.1192/j.eurpsy.2024.1520

**Published:** 2024-08-27

**Authors:** H. Ballouk, H. Boukidi, K. Taleb, S. Belbachir, A. Ouanass

**Affiliations:** Ar-Razi university psychiatric hospital, Salé, Morocco

## Abstract

**Introduction:**

Schizophrenia is characterized by a heterogeneous clinical expression. Schizophrenic symptoms fall into three main dimensions: positive, negative, and disorganized. Negative symptoms may be primary or secondary to positive symptoms.

Therapeutic compliance is essential in the management of mental illnesses and in particular schizophrenia. The associations between poor compliance and negative symptomatology are little studied even though it is found in several patients suffering from schizophrenia and is associated with a poor functional prognosis.

**Objectives:**

The objective of this study is to evaluate the link between negative symptoms and medication adherence in patients with schizophrenia.

**Methods:**

This is a cross-sectional study with a descriptive and analytical aim carried out among patients in whom a diagnosis of schizophrenia was made according to the diagnostic criteria of the DSM-5.

Data will be collected using an anonymous hetero-questionnaire including patients’ personal and sociodemographic data, as well as the negative symptoms subscale of the PANSS and Medication Adherence Rating Scale (MARS) which assesses therapeutic compliance.

**Results:**

In total, we obtained a sample of 109 patients. The median age of the population is 37 years (+/- 8.2), the age varies between 18 and 64 years. The majority of patients were single, i.e. 79.6%. On average, patients had good compliance with the MARS with a mean score of 6.3 ± 1.9 [0;10]. A negative correlation between the negative symptoms subscale of the PANSS and the MARS was found significant (p=0.003), with a moderate effect.

**Conclusions:**

This study showed that the negative signs of schizophrenia have an impact on therapeutic compliance. Therefore, it would be useful to enlarge the sample and study this association in depth in order to be able to improve these signs to ensure good care and better quality of life for these patients.

**Disclosure of Interest:**

None Declared

